# MicroRNAs and the Regulation of Tau Metabolism

**DOI:** 10.1155/2012/406561

**Published:** 2012-06-05

**Authors:** Sébastien S. Hébert, Nicolas Sergeant, Luc Buée

**Affiliations:** ^1^Axe Neurosciences, Centre de Recherche du CHUQ (CHUL), Québec, QC, Canada G1V 4G2; ^2^Département de Psychiatrie et de Neurosciences, Faculté de Médecine, Université Laval, Québec, QC, Canada G1V 0A6; ^3^Faculté de Médecine, Université Lille-Nord de France, UDSL, 59044 Lille, France; ^4^Inserm, UMR837, 59045 Lille, France

## Abstract

Abnormal regulation of tau phosphorylation and/or alternative splicing is associated with the development of a large (>20) group of neurodegenerative disorders collectively known as tauopathies, the most common being Alzheimer's disease. Despite intensive research, little is known about the molecular mechanisms that participate in the transcriptional and posttranscriptional regulation of endogenous tau, especially in neurons. Recently, we showed that mice lacking *Dicer* in the forebrain displayed progressive neurodegeneration accompanied by disease-like changes in tau phosphorylation and splicing. Dicer is a key enzyme in the biogenesis of microRNAs (miRNAs), small noncoding RNAs that function as part of the RNA-induced silencing complex (RISC) to repress gene expression at the posttranscriptional level. We identified miR-16 and miR-132 as putative endogenous modulators of neuronal tau phosphorylation and tau exon 10 splicing, respectively. Interestingly, these miRNAs have been implicated in cell survival and function, whereas changes in miR-16/132 levels correlate with tau pathology in human neurodegenerative disorders. Thus, understanding how miRNA networks influence tau metabolism and possibly other biological systems might provide important clues into the molecular causes of tauopathies, particularly the more common but less understood sporadic forms.

## 1. Introduction

The discovery of small noncoding miRNAs uncovered an intriguing additional level to the fine-tuning of the transcriptome and proteome. Since their discovery almost 20 years ago [1, 2], research has progressed considerably towards gaining a better understanding of the impact of the complex network of gene regulation by miRNAs on health and disease. This is well documented in the cancer field, for instance, where miRNAs are increasingly acknowledged as potential diagnostic and therapeutic agents [3, 4]. Like protein-coding genes, miRNA genes are transcribed from the genome mainly from RNA polymerase II (reviewed in [5]). To date, more than 1000 miRNA genes have been identified in humans and 750 in mice, some of which are specifically expressed in the brain [6–10]. In the cytoplasm, the endonuclease Dicer cleaves miRNA precursors (approximately 70 nucleotides in length) to generate mature (approximately 21 nucleotides in length) double-stranded RNAs. These are loaded as single-stranded RNAs into the RNA-induced silencing complex (RISC), composed of Argonaute (Ago) proteins, to negatively regulate gene expression, albeit some exceptions have been documented [11, 12]. This regulation is achieved through binding with imperfect complementarity mainly to the 3′ untranslated region (3′UTR) of target messenger RNAs (mRNAs), leading to translation inhibition or degradation. Both *in vitro* and *in vivo* studies have shown that alterations of a single miRNA (or miRNA family) could have profound effects on hundreds of target genes [13, 14], thus possibly implicating multiple biological pathways.

Abnormal phosphorylation and insoluble deposition of tau are observed in more than 20 neurodegenerative disorders, collectively known as tauopathies (reviewed in [15]). In Alzheimer's disease (AD), the most common tauopathy, hyperphosphorylated tau accumulates in the somatodendritic compartment of neurons, aggregates, and finally forms neurofibrillary tangles (NFTs). Other tauopathies include frontotemporal lobar degeneration (FTLD), Pick's disease, progressive supranuclear palsy (PSP), corticobasal degeneration (CBD), and progressive aphasia, all of which are characterized by neuronal and/or glial tau inclusions.

Although the exact physiological role of tau remains under scrutiny, it is proposed to function to promote microtubule assembly, stabilization, spacing, and parallel-ordered organization, which are necessary for axonal transport and neurite outgrowth (reviewed in [16]). Tau binds to several proteins (reviewed in [17]) and therefore could participate in various other paradigms, including targeting the Src kinase Fyn to dendrites [18]. In the central nervous system, tau is expressed as six isoforms, resulting from inclusion or exclusion of alternative exons 2, 3, and 10 [19] (reviewed in [20]). Mutations in or surrounding tau exon 10, leading to an imbalance in tau isoforms with 3 or 4 microtubule-binding domains, can cause familial FTLD-17. Thus, tau missplicing can cause neurodegeneration and dementia in adulthood. Changes in tau isoforms have also been observed in various other tauopathies, including PSP and Pick's disease or myotonic dystrophy (reviewed in [21]); however, the underlying mechanisms involved in abnormal tau splicing and neurodegeneration remain ill-defined. Moreover, whether splicing abnormalities function upstream or concomitantly with tau hyperphosphorylation to promote neurodegeneration remains an open debate.

To date, several groups have identified factors involved in the regulation of tau splicing. These include regulatory sequences (cis-elements) within or around tau exon 10 as well as specific regulatory proteins (trans-acting factors) (reviewed in [20]). Similarly, a number of enzymes have been proposed to regulate tau phosphorylation (reviewed in [22]). Although these studies have been insightful, they were mostly based on artificial and/or overexpression paradigms, which makes it difficult to extrapolate these observations to endogenous tau.

In 2006, Bilen et al. [23] showed a remarkable enhancement of tau-mediated cell death in Drosophila cells upon suppression of miRNA maturation. Specifically, the authors showed that retinal degeneration caused by the expression of normal or mutant (R406W) human tau *in vivo* was enhanced by loss of R3D1/loquacious, a double-stranded RNA binding protein that is required for the activity of Dcr-1 (Dicer homologue) in miRNA processing. Interestingly, upregulation of *bantam* suppressed tau-induced degeneration, suggesting that this miRNA could mitigate tau-induced neurotoxicity. However, as *bantam* is not conserved in humans, it is tempting to speculate that other miRNAs play a similar role in the mammalian brain. More recently, a neuronal miRNA, miR-128, was shown to modulate the expression of BAG2, a cochaperone potentially involved in tau degradation and aggregation in cultured COS-7 cells and in primary neurons [24]. Our recently published data indicate that conditional knockout (cKO) of *Dicer* (resulting in a global reduction in miRNA production) in the adult mouse brain results in disease-like hyperphosphorylation of endogenous tau [25]. Moreover, the *Dicer* mutant mice display changes in tau exon 10 splicing [26], as seen in various tauopathies including PSP and Pick's disease. In this paper, we highlight salient observations with regard to these studies and highlight outstanding questions related to miRNA research in tauopathies.

## 2. Tau Phosphorylation Regulation by the miR-15 Family

Tau is a phosphoprotein that contains more than 80 potential phosphorylation sites (reviewed in [27]). It is generally well accepted that tau phosphorylation is important for microtubule binding, whereas phosphorylation causes tau to detach from microtubules. Hyperphosphorylation, defined as increased phosphorylation of physiological sites and additional phosphorylation at pathological sites, characterizes insoluble aggregates of tau. However, in its unphosphorylated form, under thermal stress, tau localizes to the nucleus, where it protects DNA from double-stranded DNA damage [28]. A phosphorylated pool of tau could also localize to somatodendritic compartment as well as the dendritic spine to modulate neuronal plasticity and glutamatergic transmission [18]. Tau phosphorylation is very sensitive to intrinsic and extrinsic changes (e.g., heat, cold, stress, and starvation) [29–32]. Thus, any changes in the delicate balance between the tau kinases and phosphatases could have serious biological consequences, and the identification of these regulatory enzymes *in vivo* is of particular importance. It is noteworthy that some of those central key kinases also regulate indirectly tau splicing through phosphorylation of splicing factors [33–36]. Together, deregulation of kinase and/or phosphatase activity could be dually detrimental towards tau splicing and phosphorylation, which synergistically would promote tau aggregation.

As general posttranslational regulation of gene expression, miRNAs are potential modulators of kinase, phosphatase, and/or splicing factor expression. While studying the effects of *Dicer* loss in the brain, we observed significant changes in endogenous tau phosphorylation and splicing [26]. This was demonstrated at the RNA and protein levels using, RT-PCR, 2D electrophoresis and tau phospho-specific antibodies. Because of using the Cre-LoxP system, Dicer inactivation was limited to neurons, and in particular postmitotic pyramidal neurons. It is noteworthy that only a few studies have documented changes in endogenous tau phosphorylation *in vivo*, as most biological models express exogenous and/or mutated human tau. Remarkably, several phosphoepitopes related to disease, including serine 422, were increased in the *Dicer* mutant mice when compared with controls [25]. Unfortunately, given the rather quick lethality associated with *Dicer* loss (approximately 4 weeks), we could not determine whether tau hyperphosphorylation concurred before or after neurodegeneration. Nevertheless, these results provide a proof of concept that miRNA haploinsufficiency causes abnormal tau hyperphosphorylation and missplicing.

As stated above, several tau kinases and phosphatases have been identified, some of which are believed to contribute significantly to tau hyperphosphorylation *in vivo*. In attempt to identify such enzymes in the *Dicer* cKO mice, we performed whole-genome microarrays and western blot analyses. These experiments led to the identification of ERK1/MAPK3 (and possibly ERK2/MAPK1) as a major regulator of neuronal tau phosphorylation *in vivo*, at least in this model. In line with this observation, several ERK1-dependent epitopes were hyperphosphorylated in the *Dicer* mutant mice. Of course, several other enzymes can potentially contribute to tau phosphorylation misregulation in this and other models, and in particular disease conditions in humans. In line with this hypothesis, a number of proposed tau kinases, including GSK3*β* and JNK/MAPK8, are prone to miRNA regulation in various cell types [37, 38]. Nevertheless, it is interesting to observe that ERK phosphorylation is increased in tau-positive neurons in AD and other tauopathies [39–41]. In addition, ERK is essential for brain development and involved in neuronal death [42, 43]. Specific gene knockout mouse models are required to assess the role of these proteins in the regulation of tau phosphorylation and neurodegeneration *in vivo*.

**Figure 1:**
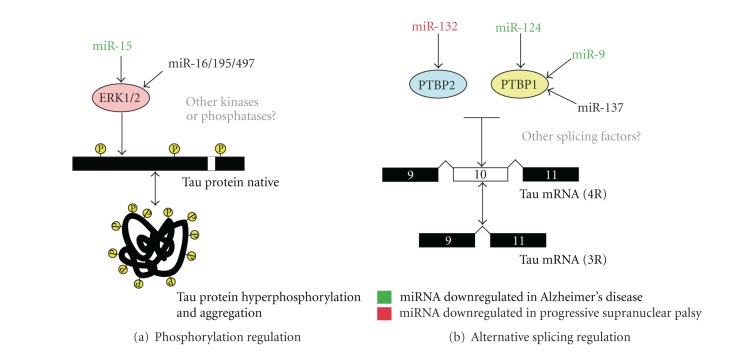
Potential role of miRNAs in tau metabolism regulation. (a, b) Two models are shown demonstrating how specific miRNAs could be involved in the regulation of tau phosphorylation (and aggregation) and/or tau exon 10 alternative splicing. Note that some miRNAs are affected in disease conditions (in green, downregulated in AD; in red, downregulated in PSP). Whether other miRNA target effectors are involved in the physiological and/or pathological regulation of tau metabolism remains to be explored. Any changes in the level or function of these miRNAs could have serious biological consequences, including tau hyperphosphorylation and aggregation and an imbalance in tau microtubule-binding repeats (encoded in tau exon 10, giving rise to either four 4R-tau or 3R-tau). Regulation of tau exon 10 splicing by PTBP1 or PTBP2 may be direct or may implicate other coregulators such as TDP-43 or PSF, both of which have been shown to either regulate PTBP2 expression or regulate PTBP splicing and repress tau exon 10 inclusion [26, 52–54].

Although it is a conceptually crude experimental approach, the *Dicer* cKO mice provide a unique and unbiased model to study global miRNA function in the brain. To identify miRNAs involved in the regulation of ERK1 (and consequently tau phosphorylation), we used several prediction programs that are available online, including TargetScan (http://targetscan.org/). This program identified a number of potential miRNA-binding sites in the 3′UTR of ERK1. Our functional assays provided the validation that several members of the miR-16 family (miR-16, -15, -195, -497) could directly modulate endogenous ERK1 and tau phosphorylation in neuronal cells *in vitro*, including rat primary neurons. Of mention, both endogenous miR-16 and tau are enriched in distal axons of sympathetic neurons [44]. Intriguingly, miR-15a and miR-15b are downregulated in AD brain and cerebrospinal fluid, respectively [25, 45, 46], providing clinical relevance for these observations. Moreover, miR-15 targets the proapoptotic protein Bcl-2, whose protein levels are increased in AD [47–49]. In addition, miR-16 overexpression could regulate APP expression *in vivo* in the mouse brain [50]. Taken together, these observations highlight the potential importance of the miR-16 family in AD development by regulating cell survival, amyloid production, and tau phosphorylation (Figure 1). Interestingly, TargetScan predicts more than 1000 human target genes for miR-16, several of which are associated with networks related to cell death, cellular organization, and molecular transport (S. S. Hébert, unpublished observations). Notably, among the high-scoring predicted targets are miRNA-processing regulators such as TNRC6B [51]. It will be interesting to see whether loss of miR-16 family members *in vivo* recapitulates, at least in past, the observed effects on ERK and, most importantly, tau phosphorylation *in vivo*.

## 3. Tau Alternative Splicing Regulation by miR-132

As discussed above, abnormal regulation of tau exon 10 splicing can cause disease. It is interesting to note that *Dicer* deficiency in the adult brain is also associated with changes in tau splicing [26]. Using a similar strategy as above (e.g., bioinformatics, microarrays, literature search, western blot analysis, etc.), we identified miR-132 and the neuronal splicing regulator PTBP2 as potential regulators of endogenous tau exon 10 splicing in neurons. These results are consistent with previous findings linking tau exon 10 splicing regulation by PTBP1 *in vitro *[55].

While not discussed in detail in our study, other miRNAs, including miR-124 and miR-9, could also regulate endogenous tau exon 10 splicing in neuronal cells by targeting specific regulatory and/or splicing factors [26]. Both miRNAs are downregulated in AD [56–58], which could have important consequences for tau metabolism, at least in certain biological contexts (Figure 1). For instance, downregulation of miR-9 is observed in the presence of exogenous A*β* in mouse primary neurons [59]. Whether this or other miRNAs function as intermediates between A*β* peptides, tau missplicing and hyperphosphorylation remain an exciting possibility. Interestingly, differential splicing of the tau transcript has also been reported in AD [60, 61].

PSP is a cause of late-onset atypical parkinsonism described by Steele et al. [62]. Dementia is also a common feature at the end stage of the disease. Neuropathologically, PSP is characterized by neuronal loss, gliosis, and NFT formation. Glial fibrillary tangles have also been described. In these patients, tau aggregates are mainly composed of tau with 4 microtubule-binding domains (4R-tau) [13, 15]. Using PSP as a model disease, we identified miR-132 to be selectively downregulated in pathological conditions. Interestingly, PTBP2 protein (but not mRNA) levels were increased in PSP patients and correlated significantly with miR-132 expression [26]. These experiments provide unprecedented molecular links among abnormal tau splicing, hyperphosphorylation, and sporadic tauopathies. Interestingly, changes in PTBP1 and PTBP2 levels, and by extension alternative splicing patterns, have been documented in human diseases, including neurodegenerative disorders [63, 64]. On the basis of these observations, it is tempting to speculate that miRNAs could contribute significantly to several aspects of tau metabolism and neuronal dysfunction in various diseases.

## 4. Outstanding Questions

Although the above-mentioned studies are interesting, many questions remain unanswered. For instance, what are the biological and clinical significance of these findings? Are other miRNAs involved in tau metabolism regulation? Can miRNAs be used as diagnostic and possibly therapeutic agents for sporadic tauopathies? Without a doubt, these and other questions will require extensive followup studies in various models, from cells to animals to humans.

## Abbreviation

3′UTR: 3′ Untranslated region.
